# High Capacity Nanocomposite Layers Based on Nanoparticles of Carbon Materials and Ruthenium Dioxide for Potassium Sensitive Electrode

**DOI:** 10.3390/ma14051308

**Published:** 2021-03-09

**Authors:** Nikola Lenar, Robert Piech, Beata Paczosa-Bator

**Affiliations:** Faculty of Materials Science and Ceramics, AGH University of Science and Technology, Mickiewicza 30, PL-30059 Krakow, Poland; nlenar@agh.edu.pl (N.L.); rpiech@agh.edu.pl (R.P.)

**Keywords:** nanocomposite mediation layers, carbon nanomaterials, ruthenium dioxide, potassium sensors, high electrical capacity

## Abstract

This work presents the new concept of designing ion-selective electrodes based on the use of new composite materials consisting of carbon nanomaterials and ruthenium dioxide. Using two different materials varying in microstructure and properties, we could obtain one material for the mediation layer that adopted features coming of both components. Ruthenium dioxide characterized by high electrical capacity and mixed electronic-ionic transduction and nano-metric carbon materials were reportedly proved to improve the properties of ion-selective electrodes. Initially, only the materials and then the final electrodes were tested in the scope of the presented work, using scanning and transmission electron microscope, contact angle microscope, and various electrochemical techniques, including electrochemical impedance spectroscopy and chronopotentiometry. The obtained results confirmed beneficial influence of the designed nanocomposites on the ion-selective electrodes’ properties. Nanosized structure, high capacity (characterized by the electrical capacitance value from approximately 5.5 mF for GR + RuO_2_ and CB + RuO_2_, up to 14 mF for NT + RuO_2_) and low hydrophilicity (represented by the contact angle from 60° for GR+RuO_2_, 80° for CB+RuO_2_, and up to 100° for NT + RuO_2_) of the mediation layer materials, allowed us to obtain water layer-free potassium-selective electrodes, characterized by rapid and stable potentiometric response in a wide range of concentrations-from 10^−1^ to 10^−6^ M K^+^.

## 1. Introduction

Since 1970s, studies over ion-selective electrodes developed for the potentiometry method focused on their modification with various electroactive materials [1,2]. The construction of ISEs is simple, as the primary type of electrodes consist of an electronic conductor (i.e., glassy carbon) and ion-selective polymeric membrane. This group of sensors is named coated-disc electrodes [3]. However, accompanying the advantages of simple construction are the drawbacks resulting from it. Indirect contact between the membrane of ionic conduction and an electronic conductor caused disruption in the charge transfer processes, which subsequently led to the aggravation of the potentiometric response (poor stability and irreproducibility of electrodes’ response) [4]. It was, therefore, concluded that there is a need for a material to be placed at the interface between an electronic and ionic conductor, and there is a constant search for these materials.

Electroactive materials that should be applied in ion-selective electrodes should fulfill a number of requirements [5]. Layer materials are said to exhibit both types of transduction—electronic and ionic, to overcome the barrier between the electronic conductor and the membrane, a high redox capacitance for obtaining stable potentiometric response [6]; hydrophobicity to eliminate the undesired water layer at the membrane–layer interface [7]; and chemical stability including the absence of any side reactions during the process of ion-to-electron transduction [8].

Amongst the studied types of materials, carbon nanomaterials, including carbon black, graphene, and carbon nanotubes, is one of the most promising [1]. Carbon nanotubes (CNTs) are characterized by a large surface-to-volume ratio, high charge-transfer capacity, hydrophobicity, and chemical stability, and were successfully applied by Crespo et al. in K^+^-selective electrodes [9]. Graphene, as a two-dimensional carbon nanomaterial, has similar characteristics to CNTs, such as a large specific surface area, fast electron-transfer capability, and excellent conductivity. Graphene (GR) was introduced into the K^+^-ISEs construction by Ping et al. [10]. Carbon black (CB), as a form of amorphous carbon, shows advantages of a low production cost, large surface area, high conductivity, and hydrophobicity, and for the first time, was used for K^+^-selective electrodes construction by Paczosa-Bator [11].

Because of their good characteristics in the context of designing ion-selective electrodes, carbon materials were combined with ruthenium dioxide to design one superior layer material, by merging the properties of both components. In this work, we present the new electroactive material that can be applied as a solid-contact layer in ion-selective electrodes that consists of carbon nanomaterials–CNTs, graphene and carbon black, and transition metal oxide–ruthenium dioxide.

As a single-component layer, ruthenium dioxide was already successfully applied in ion-selective electrodes, as presented in [12,13]. This material turned out to fulfill all requirements mentioned before for the mediation layer material, with a considerably high electrical capacitance, ensuring a stable and fast potentiometric response. Designed K^+^-selective RuO_2_-based electrodes do not exhibit the presence of water layer or any side reactions during charge-transfer processes.

The aim of this work was to combine the properties of a supercapacitor and pseudo-capacitor, i.e., carbon nanomaterials and ruthenium dioxide, respectively, in order obtain a material with a large surface area. The increase in surface area of material leads to more active sites for electrochemical reactions to occur, in the interface between layer (electrode) and electrolyte, which ensures a high electrical capacity of electrodes and subsequently their stable potentiometric response. In the results section, the properties of obtained hybrid materials were first studied, followed by their implementation into polymeric membrane-based ion-selective electrodes.

The desired application of the designed electrodes were routine analysis of potassium ions in aqueous solutions in the scope of the potentiometry method, where the potential of indicator ion-selective electrode is measured towards to a reference one [1]. Potassium is an important analyte found in a human body and in the external environment, which is responsible for water regulation and participation in enzymatic processes [14]. Routine analysis that might be conducted with the use of the potentiometric sensors presented in this work, covered determination of potassium in environmental materials (water and liquids), biological specimens (fluids), and clinical samples. The suitability of electrodes was tested during numerous potentiometric tests, with the use of standard solutions. One of the leading trends in designing new ion-selective electrodes was finding electrodes with high stability, repeatability, and reproducibility of the potential [4,6]. Fulfillment of these requirements would reduce the need for frequent calibration. Presented in the scope of this work, the electrodes are characterized by a high potential stability, therefore, they are suitable for the desired application.

## 2. Chemicals

Ruthenium dioxide in the hydrous form was purchased from Alfa Aesar (Haverhill, FL, USA). Carbon materials (CM) including Multiwalled Carbon Nanotubes (hereinafter referred to as NT), Single Layer Graphene (GR), and Printex U Carbon Black (CB) were obtained from the Nanostructured & Amorphous Materials, Inc. (Houston, TX, USA), ACS Material (Pasadena, CA, USA), and Evonik Degussa GmbH, Inorganic Materials, Frankfurt, Germany, respectively.

The membrane components—potassium ionophore I (Valinomycin), lipophilic salt-potassium tetrakis(4-chlorophenyl)borate (KTpClPB), 2-nitrophenyl octyl ether (o-NPOE), and poly(vinyl chloride) (PVC) were purchased from Sigma-Aldrich (Saint Louis, MO, USA).

Dimethylformamide (DMF) used for RuO_2_ and carbon nanomaterials dispersion and Tetrahydrofuran (THF), applied as a membrane solvent, were purchased from Sigma Aldrich (Saint Louis, MO, USA).

Chloride solutions, potassium (KCl), and sodium (NaCl) were purchased from POCH (Gliwice, Poland) and Sigma-Aldrich (Saint Louis, MO, USA), respectively. The standard K+ ions solutions used for all conducted measurements, including potentiometry, cyclic voltammetry, electrochemical impedance spectroscopy, and chronopotentiometry, were prepared from 1 M KCl standard solution. For the preparation of aqueous solutions, distilled and deionized water was used.

All chemicals were used as received, without any further purification.

## 3. Electrodes Preparation

For the purpose of the presented work, ion-selective electrodes were prepared and examined, including 3 groups of solid-contact electrodes and one coated-disc electrode used as a control (3 items per group). All electrodes were covered with potassium selective polymeric membrane of the following composition—potassium ionophore I 1.10% (*w*/*w*), KTpClPB 0.25% (*w*/*w*), o-NPOE 65.65% (*w*/*w*), and PVC 33.00% (*w*/*w*). All membrane components of total weight 0.125 g were dissolved in 1 mL of THF.

Coated-disc electrode was obtained by casting the Glassy Carbon Disc (GCD) electrode with 60 μL of membrane cocktail, after being polished on alumina slurries and cleaned ultrasonically with water and methanol. Membrane was placed onto the electrodes with the use of the dropping method. Casted electrode was left to dry in room temperature.

Solid-contact electrodes were prepared with the use of designed hybrid RuO_2_-carbon nanoparticles material as the mediation layer, placed between the electrodes’ surface and the ion-selective membrane.

Ruthenium dioxide–carbon nanoparticles materials for solid-contact layers were prepared as dispersions of 3 mg of RuO_2_ and 4 mg of carbon black, graphene, or Multi-Walled Carbon Nanotubes MWCNTs, respectively, in 1 mL of DMF.

Polished and cleaned electrodes were first covered with layer material solution in DMF and subsequently after being dried in elevated temperature, with membrane solution. All electrodes were prepared with the use of the simplest technique, i.e., drop casting. Appropriate volume of the membrane solution was dropped onto the electrodes’ disc surface from the pipette. No additional binder was required when using this technique, as the membrane’s solid particles adhere to the electrode (or mediation layer, in case of solid-contact electrodes) after solvent (THF) evaporation.

The CGD/CB + RuO_2_/K^+^-ISM group was prepared by dropping 10 μL of CB+RuO_2_ solution onto the electrode surface, CGD/GR + RuO_2_/K^+^-ISM was prepared by dropping 10 μL of GR + RuO_2_ solution, and the CGD/NT + RuO_2_/K^+^-ISM group was prepared using 15 μL of NT + RuO_2_ solution. The difference in the applied amount of the NT + RuO_2_ layer, in comparison with the other two materials, arises from the difficulties in obtaining consistent nanotubes-based layer on the electrode’s surface. Achieving homogenous NT + RuO_2_ layer required a greater volume of solution to be placed onto the electrode to ensure homogenous coverage. The amount of material solutions were selected to ensure the highest value of capacitance parameter. For greater amounts, the increase was not observed.

Both layers and membranes were casted onto electrodes, using the drop casting method, which ensured a fast and easy preparation procedure of the studied all-solid-state electrodes.

## 4. Methods

In the scope of the presented work, first, the properties of nanocomposite materials applied as mediation layers are discussed, followed by the properties of ready to use ion-selective electrodes with nanocomposite layers.

The designed ruthenium dioxide–carbon nanoparticles hybrid materials were examined using electron microscopes and contact angle microscope. Electron microscopes were implemented for the microstructure observation and included Scanning Electron Microscope (LEO 1530, Carl Zeiss, Germany) and Transmission Electron Microscope (Tecnai 20 X-TWIN, FEI, Hillsboro, OR, USA). Contact angle measurements were performed with the use of Theta Lite microscope (Biolin Scientific, Västra Frölunda, Sweden) with One Attension software (3.2 version, Attention, Colorado Springs, CO, USA), and all designed layers were studied.

Mediation layers were examined using the chronopotentiometry (Ch) method and ready-to-use electrodes with membrane were electrochemically tested, using chronopotentiometry and additionally the Electrochemical Impedance Spectroscopy (EIS). All mentioned measurements were carried out using the Autolab analyzer (PGSTAT302N, Eco Chemie, Utrecht, The Netherlands) with Nova 2.1 software. The conducted tests were performed against the Ag/AgCl reference electrode (ΩMetrohm, Herisau, Switzerland, type 6.0733.100), together with a glassy carbon rod used as auxiliary electrode, and the electrode with a particular layer was an indicator electrode. As the electrolyte, 10^−2^ M KCl was applied for all mentioned measurements.

The chronopotentiometry method was implemented for the evaluation of the electrical parameters of the studied layers and the ion-selective electrodes. According to the method presented by Bobacka et al. in [6], potential drift, resistance, and capacitance value was calculated based on the potential (E)-time (t) curve recorded during forced current flow. For the studied carbon materials-ruthenium dioxide nanocomposite layers and CM+RuO_2_-contacted electrodes, a current of 1 nA suggested in the mentioned work turned out to be insufficient, as in this condition, it was not possible to obtain stable chronopotentiograms. This current was too low to induce changes in electrodes’ potentiometric response, due to the high electrical capacity of the examined materials. To obtain a chronopotentiogram that enable evaluation of the electrical parameters of both layers and electrodes, the use of a higher current was required. The current (i) of ±100 nA (±1 nA for coated disc electrode) was applied and the potential was recorded for 60, for each sign. In the state of the current sign change, the potential jump was observed. The value of the potential jump (E) was used to calculate the material’s (electrode) resistance R = E/i. Potential drift as a measure of potential stability was calculated as the slope of potential–time curve (ΔE/Δt) and based on this value, electrical capacitance was calculated using the C = i(Δt/ΔE) equation.

Electrochemical Impedance Spectroscopy was also used for the evaluation of electrical capacity of electrodes. Impedance spectra were measured by applying a frequency from 100 kHz to 0.01 Hz, with an amplitude of 10 mV superimposed on open-circuit potential (OCP). Using the C = 1/(2 × πƒZ″) equation and the imaginary part of impedance Z″ value for the lowest frequency (ƒ = 0.01 Hz), the capacitance parameter was calculated.

All measurements conducted with the use of Autolab analyzer in a 3-electrode cell were carried out in 10^−2^ M KCl solution.

**Figure 1:**
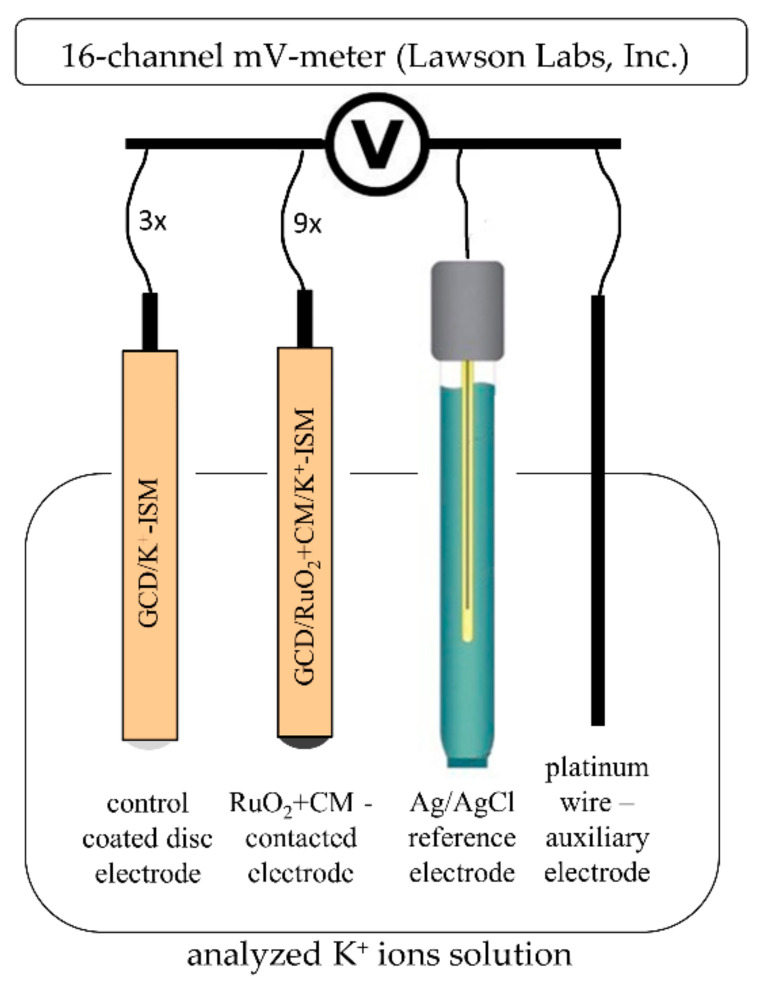
Experimental set-up for potentiometric measurements.

After examination of their electrical properties, electrodes were connected to the 16-channel mV-meter (Lawson Labs, Inc., Malvern, PA, USA) and tested in the scope of the potentiometry method. All twelve all-solid-state electrodes were examined against the Ag/AgCl reference electrode (ΩMetrohm, Herisau, Switzerland, type 6.0733.100), and in the presence of a platinum wire acting as an auxiliary electrode, as presented in Figure 1. During the potentiometric measurements, calibration curves were recorded in the solutions of K^+^ ions concentration from 10^−7^ to 10^−1^ M.

Electrodes were conditioned 1 h prior to every measurement in 10^−2^ M K^+^ ions solution (KCl), in order to saturate the ion-selective membrane with adequate amount of potassium ions.

## 5. Results

### 5.1. Morphological Characteristics of the RuO_2_–Carbon Nanoparticles Layers

**Figure 2:**
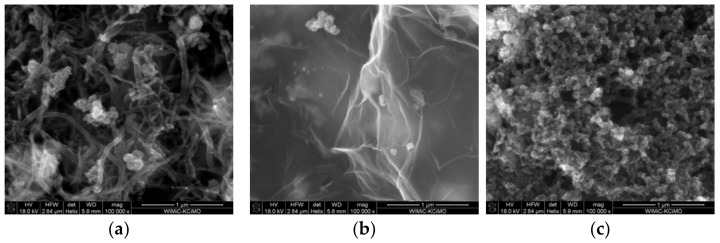
SEM scans of (**a**) NT + RuO_2_, (**b**) GR + RuO_2_, and the (**c**) CB + RuO_2_ layer.

The morphology of the layers surface was examined using Scanning Electron Microscopy. Figure 2 presents SEM scans obtained for all studied layer materials. All scans visibly presented the microstructure characteristic for carbon materials—nanotubes in Figure 2a, graphene flakes in Figure 2b, and amorphous nanoparticles of carbon black in Figure 2c. On every scan, the aggregates formed with spherical elements could be distinguished from the rest of the material components, which indicated the presence of ruthenium dioxide.

**Figure 3:**
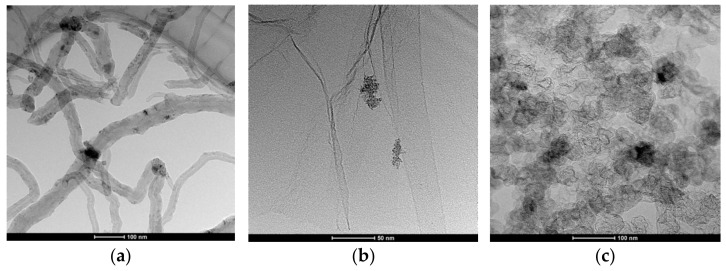
TEM scans of (**a**) NT + RuO_2_, (**b**) GR + RuO_2_, and the (**c**) CB + RuO_2_ layer.

A more thorough analysis of the layers structure was conducted with the use of Transmission Electron Microscopy (TEM). The obtained scans are presented in Figure 3.

Contrast black spots visible against the carbon materials structure (tubes—Figure 3a, flakes—Figure 3b, and amorphous spherical particles—Figure 3c of nanotubes (NT), graphene (GR), and carbon black (CB), respectively) are the ruthenium dioxide nanoparticles.

As can be seen, oxide particles effectively mix with, or adhere to carbon materials, elevating the surface area of the material itself.

Both performed analysis (with SEM and TEM microscope) confirmed the nanometric size of ruthenium dioxide particles, which in association with the nanometric elements of the carbon material structure, ensured a high surface area desired for achieving a high electrical capacitance of the material. This is relevant from the analytical point of view, as a high electrical capacitance of material for the mediation layer, enabled us to obtain a stable potentiometric response of ion-selective electrodes.

### 5.2. Wettability

After removing the aqueous solution from the ion-selective electrodes’ construction and replacing it with a solid material layer, the problem of absorbing water from the analyzed solutions through the membrane occurred. The water layer that formed between the ion-selective membrane and the electrode’s surface, not only caused delimitation of the membrane but also led to a drift of the electrode’s potentiometric response (which is explained in detail in Section 5.6.—Water Layer Test). To prevent the formation of water film, the hydrophobic mediation layer, which simultaneously exhibits ion-to-electron transduction properties, was placed between the electronic conductor and polymeric membrane.

**Figure 4:**
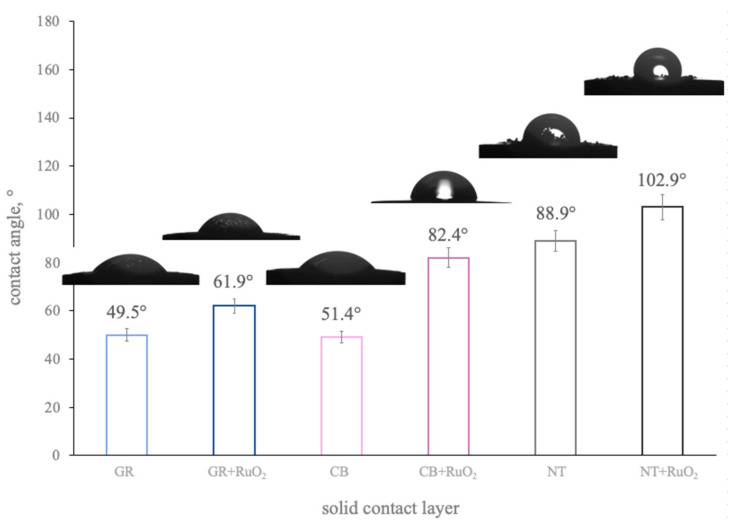
Contact angles of the studied layers; from left—graphene, GR + RuO_2_ composite, carbon black, CB + RuO_2_ composite, nanotubes, and NT + RuO_2_ composite.

The wetting properties of material (its hydrophilicity or hydrophobicity) were characterized by the contact angle value. In order to determine the contact angle values ascribed to the designed composite CM + RuO_2_ materials, the wettability test was conducted using the Theta Lite contact angle microscope by Biolin Scientific. The water drop was released from the syringe onto the tested layer (covering the GCD electrode), and the contact angle was measured using the One Attension software. The wettability properties of the composite CM + RuO_2_ layers were compared with those of the carbon nanomaterials applied separately. The results are presented in Figure 4.

As shown, all composite materials turned out to exhibit low hydrophilic/hydrophobic properties with a contact angle of 62° for the GR+RuO_2_ layer, 82° for the CB + RuO_2_ layer, and the highest value of 103° was obtained for the NT + RuO_2_ layer. Contrary to the RuO_2_-based materials, single-type carbon materials were characterized by lower contact angle values, hence, were more hydrophilic. For the GR layer, the contact angle was the lowest (less than 50°) and similar to graphene, the carbon black layer also turned out to be less hydrophobic (contact angle of approximately 51.5°). For the NT layer, the contact angle was equal to 89°. Contact angle values obtained for the nanocomposite layers were also significantly higher than the contact angle observed for the ruthenium dioxide layer, which, as reported in [15], equaled to 17°.

Based on the obtained results, it could be concluded that the addition of ruthenium dioxide to carbon materials beneficially influenced their wettability properties in the context of designing all-solid-state ion-selective electrodes, where high contact angles are desired. This feature is believed to prevent the occurrence of the aqueous layer that tends to form under the ion-selective membrane, causing deterioration of the mechanical durability and analytical parameters of the electrodes. As presented in the literature [2,16], hydrophobicity of the material for the mediation layer was preferred for the optimization of ion-selective electrode performance (to ensure a stable potentiometric response of electrodes), which is proved and described in more detail in this work, in the Water Layer Test section.

### 5.3. Electrochemical Characteristics of the RuO_2_–Carbon Nanoparticle Layers

**Figure 5:**
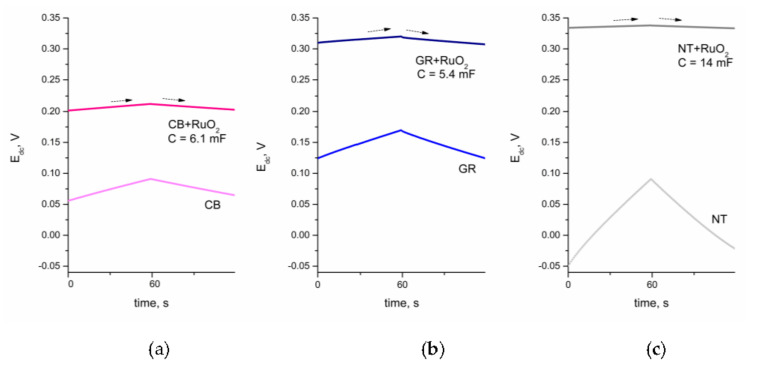
Chronopotentiograms of (**a**) CB-based, (**b**) GR-based, and (**c**) NT-based layers with (top curves) and without (bottom curves) the presence of ruthenium dioxide.

Electrical behavior of the studied solid-contact layers was evaluated using the chronopotentiometry method and the potential response was recorded as the current (of current value 100 nA) that passed through the measuring cell. Due to the use of this analytical technique, it was possible to estimate the capacitance value characterizing every studied layer. The obtained curves were compared to those recorded for the single-type layers, only consisting of the selected carbon material—carbon black, graphene, and nanotubes. Figure 5 presents chronopotentiograms of all tested layers, with an arrow pointing the moment of the current sing change (from positive to negative). Top curves presented on figures correspond to the composite layers, while the bottom ones were recorded for single type carbon material layers. It could be concluded upon result collection that the presence of ruthenium dioxide in the layer material, affected the electrochemical behavior of electrodes covered with a certain layer.

For carbon black, the capacitance value was of 2.0 ± 0.3 mF for the CB layer and 6.1 ± 0.4 mF for the hybrid CB + RuO_2_ layer. For graphene, the value was 1.32 ± 0.02 mF for the GR layer and 5.4 ± 0.3 mF for the hybrid GR + RuO_2_ layer. For carbon nanotubes, the values were 0.48 ± 0.03 mF for the NT layer and 14.0 ± 0.8 mF for the hybrid NT + RuO_2_ layer. The higher the capacitance value (the highest value obtained for NT + RuO_2_), the better was the stability of the potential response in the presence of current flow (consequently, there was a nearly flat response of the NT + RuO_2_-covered electrode).

### 5.4. Electrochemical Characteristics of the RuO_2_–Carbon Nanoparticles Electrodes

Subsequently, K^+^-selective electrodes were prepared, as described in the Electrodes Preparation section, by casting the obtained earlier layers with an ion-selective membrane cocktail. Electrodes were characterized with chronopotentiometry and EIS methods.

For the chronopotentiometry method, a constant current of 100 nA was used for the examination of CM + RuO_2_-based solid-contact electrodes and a current of 1 nA was used for examination of the coated-disc electrode, which was used in this study as a control electrode. Applying this electrochemical technique, allowed us to obtain values of electrical properties like electrical capacitance, resistance, and potential drift of the designed electrodes. The results calculated on the basis of equations presented in the Methods section are presented in Table 1.

**Figure 6:**
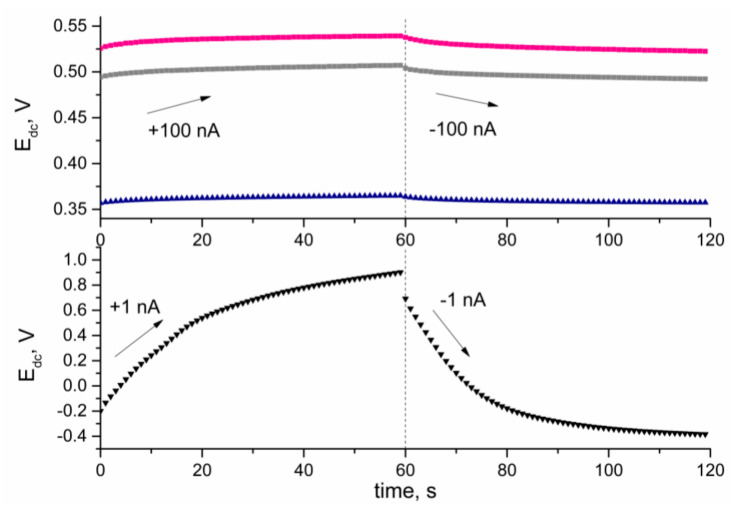
Chronopotentiograms of GC/CB+RuO_2_/K^+^-ISM (■), GC/NT+RuO_2_/K^+^-ISM (●), GC/GR+RuO_2_/K^+^-ISM (▲), and GC/K^+^-ISM (▼) electrodes.

The obtained chronopotentiograms are presented in Figure 6. First 60 s of the casting step was recorded for the positive sign current, and the following step was recorded for the negative sign current. Between both steps, a potential jump was observed, which was subsequently used for calculating the resistance value. The capacitance value was calculated for the linear part of the recorded chronopotentiograms.

As presented, in contrast to the control coated-disc electrode, the designed solid contact electrodes exhibited significantly enhanced electrical properties. For the ion-selective electrodes, high electrical capacities and low resistance and potential drift were desired for obtaining robust potentiometric sensors. Applying the CM + RuO_2_ layers allowed us to elevate the capacitance value, approximately 1000 times, and to decrease the resistance value hundredfold, in comparison to the electrode without a solid-contact layer. The potential drift was also of a much lower value with a potential change of approximately 0.2 mV per second recorded for the solid-contact electrodes, contrary to 18 mV per second for the GCD/K^+^-ISM electrode.

**Figure 7:**
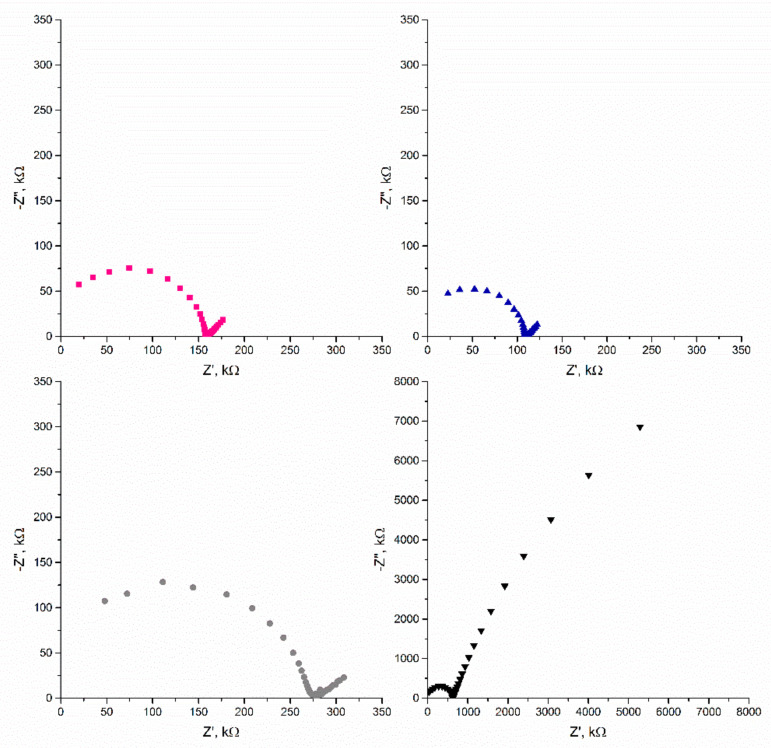
EIS curves of the GC/CB + RuO_2_/K^+^-ISM (■), GC/NT + RuO_2_/K^+^-ISM (●), GC/GR + RuO_2_/K^+^-ISM (▲), and GC/K^+^-ISM (▼) electrodes.

The other technique used for electrode characterization was electrochemical impedance spectroscopy. With this technique, the most common way to present results is the Nyquist plot, on which the imaginary part of impedance (Z″) is plotted as a function of the real impedance (Z′). The Nyquist plots for all studied electrodes are presented in Figure 7. The capacitance value could be calculated for the low frequencies (in this case ƒ = 0.01 Hz) with the use of the equation presented in the Methods section, and equaled to 869, 697, 1252, and 2.32 μF for the GC/CB + RuO_2_/K^+^-ISM, GC/NT + RuO_2_/K^+^-ISM, GC/GR + RuO_2_/K^+^-ISM, and GC/K^+^-ISM electrodes, respectively.

Both electrochemical techniques applied for testing the electrical capacitance parameter proved that despite the graphene-based layer being characterized by the lowest electrical capacitance itself (5.4 mF), after covering the electrode with an ion-selective membrane, the capacitance of the GC/GR + RuO_2_/K^+^-ISM electrode turned out to be the highest of all tested groups (2.6 mF and 1.25 mF obtained using chronopotentiometry and EIS, respectively). Although the electrical capacity of the other two materials (NT + RuO_2_ and CB + RuO_2_) was higher, the presence of the ion-selective membrane significantly decreased the capacitance value of electrodes, characterized by approximately 1 mF capacitance, given by the chronopotentiometry method and several hundred μF obtained using EIS.

Unlike the case of the mediation layers (the highest capacitance attributed to the NT + RuO_2_ layer), when examining electrodes covered with the ion-selective membrane, the best electrical parameters were obtained for the GC/GR + RuO_2_/K^+^-ISM group. Literature data revealed [6] that the recorded capacitance of solid contacts in ISEs is usually significantly lower than the capacitance of the same material and the same quantity, but in the absence of the ion-selective membrane. The effect of decreased capacitance value after applying polymeric membrane onto the solid-contact layer was widely described by Pławińska et al. in [17]. The phenomenon of capacitance decrease results from the limited amount of mobile ions, either in the mediation layer or in the membrane on the layer/membrane interface, which could be transferred across this interface. Based on this theory, it might be concluded that the studied nanocomposites are characterized by different amounts of mobile ions, therefore the capacitance value decrease after applying the potassium membrane was also different for each material. This suggests that the highest number of ions could be found in the GR + RuO_2_ material.

Electrical capacitance, being a measure of stability of the electrode potential under the influence of a small external current, is the main quantitative parameter characterizing all-solid-state ion-selective electrodes. The values obtained for the CM + RuO_2_-contacted electrodes were compared with the solid-contact electrodes, prepared with the use of other composite materials presented so far in literature.

The capacitance values obtained for other composite materials containing carbon nanotubes (with AuCu nanoparticles and conducting polymers) were as follows—54 μF, 83 μF, and 30 μF for MWCNTs-AuCuNPs [18], PEDOT-MWCNTs [19], and POT-MWCNTs [16], respectively. It could, therefore, be concluded that the RuO_2_-MWCNTs nanocomposite presented in this work exhibited a considerably high capacitance value (1045 μF), in comparison to other designed composites.

For electrodes with other graphene composite material–graphene/tetrathiafulvalene (TTF(NO_3_)-GR), the capacitance value was of 1180 μF [20], which was twice less as compared to the GR + RuO_2_–based electrodes. Another graphene composite reported in the literature was Tetracyanoquinodimethane–based composite TCNQ-GR and conducting polymer-based composite PANI-GR, for which the capacitance value was of 284 μF [21] and 11 μF [22], respectively.

Extremely high capacitance values were obtained for the carbon black–organic molecules composites–TTFCl-CB (2800 μF), presented by Pięk et al. in [23], and for TCNQ/NaTCNQ (2150 μF) presented by Hu et al. in [24].

For the platinum nanoparticles–carbon materials–contacted potassium selective electrodes capacitance values were as follows—248 μF and 154 μF for PtNPs-CB [25] and PtNPs-GR [26], respectively, which was considerably lower in comparison to electrodes with ruthenium dioxide–carbon material layers.

In our previous works, electrical capacitance presented for RuO_2_-contacted K^+^-selective electrodes equaled to 1070 μF [13], while for the RuO_2_–POT composite, we received a value of 1167 μF [15]. Designing the RuO_2_–carbon nanomaterials composites allowed us to increase the capacitance for potassium selective electrodes to 2577 μF (with the use of the GR + RuO_2_ mediation layer).

Other composite materials applied so far as solid-contact layers in ASS electrodes included, for example, TiO_2_-PANI with a capacitance value of 8 μF [27] and POT-MoS_2_ (526 μF) [28].

To summarize, taking into consideration the data presented above, the capacitance value characterizing the GR+RuO_2_ nanocomposite-contacted electrode was, to the best of our knowledge, one of the highest capacitance values reported for all-solid-state electrodes.

### 5.5. Potentiometric Response

**Figure 8:**
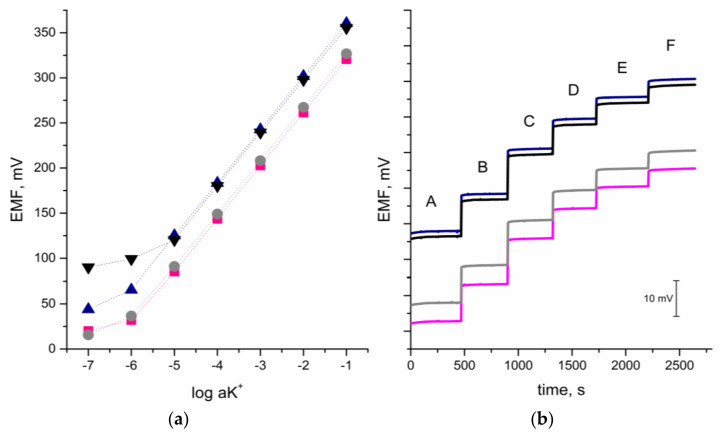
Exemplary potentiometric response of GC/CB + RuO_2_/K^+^-ISM (■), GC/NT + RuO_2_/K^+^-ISM (●), GC/GR + RuO_2_/K^+^-ISM (▲), and GC/K^+^-ISM (▼) electrodes. (**a**)—tested for standard K^+^ solutions from 10^−6^ to 10^−1^ M, (**b**)—tested for standard solutions between 10^−3^ and 10^−2^ M (A—0.001 M, B—0.002 M, C—0.004, D—0.006 and E—0.008 M, F—0.01 M).

Electrochemical tests conducted to examine and define the studied layers were followed by potentiometric studies to confirm the beneficial impact of the presence of CM + RuO_2_ layers. The calibration test was performed 3 days in a row, after 24, 48, and 72 h of electrode conditioning in the 10^−2^ M KCl solution, and the electromotive force was measured in the KCl standard solutions of concentration ranging from 10^−7^ to 10^−1^ M. The exemplary potentiometric response after 24 h of conditioning is presented in Figure 8a. Based on the obtained results, the linear range was estimated in 10^−1^ to 10^−6^ M for the CM + RuO_2_-contacted electrodes, in contrast to the 10^−1^ to 10^−5^ M range achieved by the coated-disc electrode. In the mentioned spectrum of potassium ions concentration, the studied electrodes exhibited near-Nernstian response, with a slope of calibration curve equal to 58.03, 58.25, and 58.95 for the GC/CB + RuO_2_/K^+^-ISM, GC/NT + RuO_2_/K^+^-ISM, and GC/GR + RuO_2_/K^+^-ISM electrodes, respectively. A similar potentiometric response was recognized for the RuO_2_-contacted electrodes, for which the near-Nernstain response (57.94 mV/pK) was observed in the same 10^−1^ to 10^−6^ M K^+^ ions range [13]. For the coated-disc electrode, the slope was 58.76 mV/pK.

Based on the results collected in a period of 3 days, it was possible to determine the repeatability of the designed electrodes. Standard deviation of the potential measured for the potassium ions concentrations was calculated for each electrode and each standard solution. Solid contact CM + RuO_2_–based electrodes exhibited a great repeatability over 3 days of calibration. The potential SD value for potassium ions concentrations from 10^−5^ to 10^−1^ M was no more than 0.35, 0.25, and 0.30 mV, for the GC/CB + RuO_2_/K^+^-ISM, GC/NT + RuO_2_/K^+^-ISM, and GC/GR + RuO_2_/K^+^-ISM electrodes, respectively, and about 0.5–1.5 mV for the lower potassium concentrations. For comparison, in the case of the coated disc electrode without a composite layer, the standard deviation values of the measured potentials were about 5 mV for potassium ions concentration from 10^−4^ to 10^−1^ M, and up to 10 mV for the 10^−6^ and 10^−7^ M K^+^ solution.

Reproducibility of the electrodes was evaluated for each group of solid-contact electrodes, based on their standard potential value (E_0_). As the quantitative parameter of reproducibility, standard deviation values were calculated from the values obtained for three items representing each group (*n* = 3), after 24 h of conditioning. The obtained values of average standard potential were as follows—375 ± 2, 382 ± 4, and 420 ± 1 mV, for the GC/CB + RuO_2_/K^+^-ISM, GC/NT + RuO_2_/K^+^-ISM, and GC/GR + RuO_2_/K^+^-ISM group, respectively. Taking into consideration the obtained SD values, the best reproducibility of the potentiometric response could be attributed to the GC/GR + RuO_2_/K^+^-ISM group, in which the convergence of the standard potential of all electrodes representing the group was the best.

Figure 8b displays the electrochemical behavior of electrodes, while changing the K^+^ ions concentration gradually from 10^−3^ to 10^−2^ M (through 0.002, 0.004, 0.006, and 0.008 M). Both equilibrium value and the process of achieving the final value of potential was presented. As shown, the response of all studied electrodes was stable almost immediately after exchanging the potassium ion solution in the studied concentration range.

**Figure 9:**
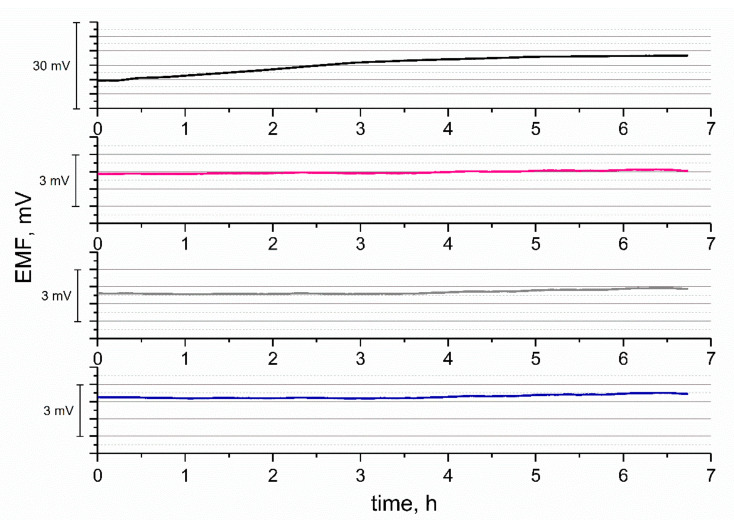
Stability test—potentiometric response of GC/CB+RuO_2_/K^+^-ISM (pink line), GC/NT+RuO_2_/K^+^-ISM (grey line), GC/GR+RuO_2_/K^+^-ISM (blue line), and GC/K^+^-ISM (black line) electrodes, over 6 h, conducted in 10^−2^ M K^+^ solution.

The stability of the designed electrodes was also tested through the longer period of time (6 h). The EMF was recorded in 10^−2^ M K^+^ solution and presented in Figure 9 as a function of time.

As presented, for the coated wire GC/K^+^-ISM electrode, the potential drift of approximately 1 mV/h was observed. For the CM + RuO_2_-contacted electrodes, the potential fluctuations were smaller, with the best potential stability exhibited by the GC/GR + RuO_2_/K^+^-ISM electrode. This good performance of graphene-based electrode could be explained by its high electrical capacitance (as presented in the electrochemical characteristic section), as for this group of electrodes, the potential drift during the chronopotentiometric test was also of the lowest value. For single-component ruthenium dioxide-contacted electrodes, potential stability was also remarkable (with only 0.085 mV potential change per hour) [13].

### 5.6. Water Layer Test

The last test conducted on the designed potassium selective electrodes was the water layer test. During the course of numerous potentiometric measurements, electrodes tend to absorb water from aqueous solutions, through the ion-selective membrane. Absorbed water form a thin layer between membrane and electronic conductor (in case of coated-disc electrodes) or mediation layer (in the solid-contact electrodes), which is a cause of the potential drift of electrode response and might result in deterioration of membrane adherence. It is, therefore, important to prevent the formation of such layers already at the stage of designing ion-selective electrodes. Water uptake could be limited by introducing the hydrophobic material into electrode construction as mediation layers.

In order to investigate the ability of the studied carbon materials–ruthenium dioxide layers to eliminate the formation of aqueous film, the water layer test was conducted according to the procedure presented by Fibbioli et al. in [29] and Guzinski et al. in [30].

**Figure 10:**
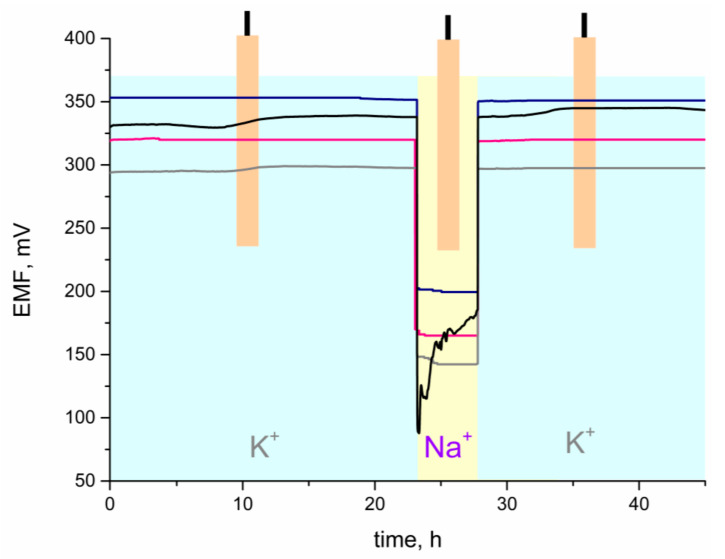
Water layer test for GC/CB + RuO_2_/K^+^-ISM (pink line), GC/NT + RuO_2_/K^+^-ISM (grey line), GC/GR + RuO_2_/K^+^-ISM (blue line), and GC/K^+^-ISM (black line) electrodes.

During this test, the potential was recorded, while the solution of primary potassium ions was exchanged with the solution of interfering ions (sodium ions) and the potential drift was monitored. For the purpose of the water layer test, the electrodes were placed into 10^−2^ M KCl solution for 24 h, then KCl was exchanged into 10^−2^ M NaCl solution to examine the potential drift, and after 5 h, this was exchanged back to the primary ion solution, to examine the stability of potentiometric response. The interpretation of the test’s results relied on the examination of potential drift, after exchanging the primary ions into interfering ions. If the drift occurred and the stabilization of the potential was slow, it could be concluded that the water film formed under an ion-selective membrane. On the other hand, if the drift was not observed, it implied that the water film did not exist. Potentiometric response of all groups of studied electrodes—coated disc and CM + RuO_2_-contacted electrodes was recorded, with the time and results presented in Figure 10.

As can be seen, at the beginning of measurement, when electrodes were placed into the KCl solution, it took longer for the coated-disc electrode to reach the stable potentiometric response and a potential drift was observed. After exchanging the solution back from sodium chloride into potassium chloride, an analogous situation was observed and a more substantial potential drift was observed for the non-modified electrode. Electrodes with carbon materials–ruthenium dioxide composite layers exhibited more stable potentiometric response, before and after contacting the sodium ions solution. It could therefore be concluded that the presence of the designed composite layers beneficially affected the potentiometric response of ion-selective electrodes and prevented the formation of water layer under a polymeric membrane. Thanks to the CM + RuO_2_ mediation layers, no potential drift was observed, which proved that the water film was not formed and the solid-contact electrodes performed better and lived longer than the coated-disc electrode.

The designed solid-contact electrodes exhibited great stability and reproducibility of potential during a 9-months lasting test. The deterioration of potentiometric response was not observed with time. Thanks to the absence of the water layer, the obtained CM + RuO_2_-based electrodes were durable, and the membranes adhered properly to the applied layers through the period of long-lasting measurements.

## 6. Conclusions

Searching for new electroactive materials for mediation layers in ion-selective electrodes was the subject of interest for many years. In the scope of this work, three different layers were tested—NT + RuO_2_, GR + RuO_2_, and CB + RuO_2_ composite materials, and their properties were compared with single-type carbon nanomaterials layers. The proposed solution based on introducing ruthenium dioxide and carbon nanomaterials into one layer allowed us to obtain materials characterized by extremely high electrical capacitance of up to 14 mF for the NT+ RuO_2_ composite layer. The addition of ceramic dioxide caused the increase of capacitance parameter value for all tested carbon nanomaterials. Consequently, elevating the capacitance value, beneficially influenced the potentiometric response of electrodes, which was fast, reversible, and stable with the time of measurement for all tested electrodes. Carbon nanomaterials–ruthenium dioxide-based electrodes exhibited near-Nernstian response, in wide concentrations, ranging from 10^−6^ to 10^−1^ M K^+^ ions. Another feature of the designed layers was low hydrophilicity, which ensured the lack of water layer formed between the ion-selective membrane and the electrode material, which prevented the occurrence of potential drift. In addition to excellent electrical and analytical properties, competitive to other solutions presented in the literature for solid-contact ISEs, the proposed method of producing sensors was fast and easy. The presented sensors could be readily obtained using basic laboratory equipment and the mentioned components.

## Figures and Tables

**Table 1:** Electrical parameters of studied electrodes (*n* = 3) obtained from the chronopotentiometric measurements.

Group of Electrodes	Resistance ± SD (kΩ)	Potential Drift ± SD (mV/s)	Capacitance ± SD (μF)
GCD/K^+^-ISM	1480 ± 60	18 ± 2	1.42 ± 0.06
GC/CB+RuO_2_/K^+^-ISM	7.63 ± 0.03	0.24 ± 0.02	1080 ± 22
GC/NT+RuO_2_/K^+^-ISM	15.26 ± 0.12	0.203 ± 0.009	1045 ± 28
GC/GR+RuO_2_/K^+^-ISM	5.64 ± 0.02	0.118 ± 0.007	2577 ± 35

## Data Availability

Data sharing is not applicable to this article.
